# Immobilization, Regiospecificity Characterization and Application of *Aspergillus oryzae* Lipase in the Enzymatic Synthesis of the Structured Lipid 1,3-Dioleoyl-2-Palmitoylglycerol

**DOI:** 10.1371/journal.pone.0133857

**Published:** 2015-07-28

**Authors:** Haiying Cai, Yang Li, Minjie Zhao, Guanwen Fu, Jia Lai, Fengqin Feng

**Affiliations:** 1 College of Biosystems Engineering and Food Science, Zhejiang University, Hangzhou, China; 2 Zhejiang Key Laboratory for Agro-Food Processing, Zhejiang University, Hangzhou, China; 3 Hangzhou Kangyuan Food Technology Co., Ltd, Hangzhou, China; 4 Fuli Institute of Food Science, Zhejiang University, Hangzhou, China; Virginia Tech, UNITED STATES

## Abstract

The enzymatic synthesis of 1,3-dioleoyl-2-palmitoylglycerol (OPO), one of the main components of human milk fats, has been hindered by the relatively high cost of sn-1,3-specific lipases and the deficiency in biocatalyst stability. The sn-1,3-specific lipase from *Aspergillus oryzae* (AOL) is highly and efficiently immobilized with the polystyrene-based hydrophobic resin D3520, with a significant 49.54-fold increase in specific lipase activity compared with the AOL powder in catalyzing the synthesis of OPO through the acidolysis between palm stearin and oleic acid (OA). The optimal immobilization conditions were investigated, including time course, initial protein concentration and solution pH. The sn-1,3 specificity of lipases under different immobilization conditions was evaluated and identified as positively associated with the lipase activity, and the pH of the immobilization solution influenced the regiospecificity and synthetic activity of these lipases. Immobilized AOL D3520, as the biocatalyst, was used for the enzymatic synthesis of the structured lipid OPO through the acidolysis between palm stearin and OA. The following conditions were optimized for the synthesis of structured lipid OPO: 65 °C temperature; 1:8 substrate molar ratio between palm stearin and OA; 8% (w/w) enzyme load; 3.5% water content of the immobilized lipase; and 1 h reaction time. Under these conditions, highly efficient C52 production (45.65%) was achieved, with a tripalmitin content of 2.75% and a sn-2 palmitic acid (PA) proportion of 55.08% in the system.

## Introduction

Human milk fat (HMF), comprising only 3–5% of human milk, is one of the main sources of nutrients and energy for infants, providing approximately 50–60% of the dietary energy required for infants[1]. HMF primarily exists as triacylglycerols (TAGs>98%), on which the fatty acid (FA) displays a regiospecific distribution that palmitic acid (PA>60%), along with other saturated FAs, preferably locates at the sn-2 position of the backbone, whereas the unsaturated FAs, predominantly oleic acid (OA), are primarily located at the sn-1,3 positions[2]. This characteristic plays a critical role in facilitating fat absorption in infants[3]. Therefore, 1,3-dioleoyl-2-palmitoylglycerol (OPO), as one of the main components of the HMF, has attracted much attention in the panorama of an increasing demand for human milk fat substitutes (HMFS) and has been successfully used as a nutrition frontier to alleviate the problems resulting from the intake of infant formulas produced from vegetable oils, such as the loss of energy and calcium, constipation, abdominal pain and intestinal obstruction[4, 5].

The enzymatic synthesis of OPO involves the reaction between TAGs or 2-monoacylglycerol (2-MAG) rich in PA at the sn-2 position and acyl donors (mainly OA, and its methyl ester) catalyzed through sn-1,3-specific lipases. Currently, tripalmitin, fractionated palm stearin, butter fat, and lard[6] have been demonstrated as suitable PA donors for OPO production, and some of these fats have been commercially used to manufacture human milk fat substitutes through the enzymatic synthesis[6]. Unfortunately, the enzymatic synthesis of OPO suffers from potential acyl migration, resulting in the quality deterioration of products[7]. In addition, the prosperity of the structured lipids in the food industry has been hindered as a result of the relatively high cost of sn-1,3-specific lipases and the deficiency in the stability of the synthesis reactions, primarily reflecting the sensitivity of the biocatalyst in the harsh reaction environment. The adoption of novel stable regiospecific lipases and immobilization treatments might be promising to alleviate these problems. Several sn-1,3-specific lipases have been assessed for the enzymatic synthesis of OPO, among which the lipases from *Rhizomucor miehei* and *Thermomyces lanuginosa* have received much attention, reflecting the distinguished sn-1,3 specificity and thermostability of the immobilized forms of these enzymes in organic solvent[8, 9]. To our knowledge, there are no studies examining the lipase from *Aspergillus oryzae* (AOL) as a biocatalyst for OPO synthesis, although AOL L3 has been characterized as sn-1,3 specific[10]. In addition, the FDA has classified AOL as GRAS (generally regarded as safe), and this enzyme shows increased resistance to organic solvent media[11, 12]. Therefore, AOL is a potential alternative to the current commercial sn-1,3-specific lipase for the enzymatic synthesis of structured lipids.

Immobilization is particularly critical for lipase application, not only because this characteristic contributes to the mass transfer, lipase stability, enzyme recycling and reuse, product purification and other important features that benefit enzyme immobilization[13–15], but also because immobilization might significantly increase catalytic activity, particularly in a water-free system, likely reflecting the variation of the lipase conformation to the open active form[16, 17]. Lipase immobilization based on adsorption, rather than other typical immobilization methods, such as entrapment, covalent coupling or cross-linking, has been preferably adopted, primarily reflecting the simple manipulation and significant enhancement of lipase activity[18]. The adsorption of lipase into supports requires adequate interactions, most commonly hydrophobic and in some cases ionic interactions, between these components[19]. Consequently, a variety of hydrophobic supports, such as polypropylene-based supports[20] and styrene-divinylbencene resin, have been introduced as carrier candidates for lipase immobilization[18]. Novozyme 435 (Novozymes, Denmark), frequently used in food and pharmaceutical industries, is a commercial lipase preparation of *Candida antarctica* lipase immobilized with Lewatit, a methacrylate-styrenedivinylbenzene resin carrier. Polystyrene (PS) macroporous resin has also been used to immobilize the lipase from *Burkholderia cepacia*, and this resin has been successfully demonstrated to enhance lipase activity together with bioimprinting and interfacial activation[21]. In addition, as a common raw material in industry, macroporous PS has been increasingly used in lipase immobilization, as this support has enormous sources and low costs.

The aim of the present study is intended to immobilize the sn-1,3-specific lipase AOL into suitable resin supports through an adsorption mechanism to obtain highly efficient industrial biocatalysts for the enzymatic synthesis of the structured lipid OPO. Seven non- or low-polar macroporous resins and four ion-exchange macroporous resins were investigated as potential supports. Furthermore, the acidolysis reactions between palm stearin and OA were performed and optimized for the enhanced production of OPO. Some influencing factors on the regiospecificity of lipase were investigated. The results of the present study provide the first evidence that the sn-1,3 specificity of lipase should be evaluated along with lipase activity, and pH was found to significantly alter the regiospecificity.

## Materials and Methods

### Materials

AOL L03 (*Aspergillus oryzae* lipase powder) was purchased from Leveking Bioengineering Co.,Ltd. (Shenzhen, China). Lipozyme RM IM (*Rhizomucor miehei* immobilized on an ion-exchange resin), Lipozyme TL IM (*Thermomyces lanuginosa* immobilized on silica gel) and porcine pancreatic lipase (PPL) were purchased from Novozymes A/S (Bagsvaerd, Denmark). Macroporous resins AB-8, D3520, NKA were acquired from the Chemical Plant of NanKai University (Tianjing, China). Macroporous resins SD300, SD600, DM11, DM130, anion-exchange macroporous resins D354 FD, D314 FD, D318, and cation-exchange resin C258 FD were purchased from Zhejiang Zhengguang industrial Co., Ltd. (Hangzhou, China). Table 1 comparitively describes the properties of these materials. The standards of tripalmitin, C52 compound (primarily OPO and OPO isomeric compounds), diolein (mixed isomers) and monoolein were from Sigma-Aldrich (St Louis, USA). Palm stearin (tripalmitin 48.69%, C52 13.42% and sn-2 PA 27.18%) and OA (82.5%) were obtained from Xinshili Food Science Co., Ltd. (Nanjing, China) and Yihai Kerry Fine Chemical Co., Ltd (Lianyungang, China), respectively. Other reagents used were either HPLC or analytical reagent grades and obtained from various sources.

**Table 1:** Carriers for immobilization of AOL and their properties.

Name	Matrix	Resin type	Water content (%)	Mean diameter (nm)	Mean pore diameter (nm)	Specific area (m^2^/g)
AB-8	PS	Low polar	60–70	300–1250	13–14	480–520
D3520	PS	Non-polar	70–80	300–1250	8.5–9.0	480–520
NKA	PS	Non-polar	62–72	300–1250	20–22	570–590
DM11	PS	Non-polar	60–70	300–1250	9–10	200–250
DM130	PS	Low polar	65–75	300–1250	8–9	200–300
SD300	PS	Low polar	55–65	300–1250	2–3	800–1000
SD600	PS	Low polar	55–65	300–1250	2–3	1000–1200
Name	Matrix	Resin type	Water content (%)	Mean diameter (nm)	Bulk density (g/ml)	True density (g/ml)
D354 FD	PS	Anion-exchange with free amine	48.0–58.0	315–1250	0.65–0.75	1.02–1.08
D314 FD	Poly(acrylic acid)	Anion-exchange with free amine	60.0–65.0	315–1250	0.65–0.75	1.06–1.10
D318	Poly(acrylic acid)	Anion-exchange with free amine	50.0–60.0	315–1250	0.65–0.75	1.02–1.10
C258 FD	Poly(acrylic acid)	Hydrogen cation-exchange	45.0–55.0	315–1250	0.72–0.82	1.14–1.22

### AOL L03 immobilization

To screen the suitable carriers for AOL L03 immobilization, 11 resins were tested. Non-polar and minor polar macroporous resins AB-8, D3520, NKA, SD300, SD600, DM11, and DM130 were successively prewetted in 10% NaCl, 95% ethanol, 5% HCl and 2% NaOH for 4 h and washed with deionized water. The ion-exchange macroporous resins C258 FD, D354 FD, D314 FD and D318 were successively treated with deionized water, 6% NaCl, 4% HCl and 4% NaOH for 4 h and washed with deionized water. Approximately 3 g of AOL L03 powder was dissolved and mixed into 100 ml of glycine-NaOH buffer solution (0.1 M, pH 9.0), and subsequently centrifuged at 2000 rpm for 30 min at room temperature. The protein concentration in the supernatant was analyzed using the Bradford method [22], and the supernatant was subsequently used for the following lipase immobilization. A total of 20 ml of enzyme solution (30 mg/ml) and 4 g of prepared support were added into the flask and shaken at 200 rpm at 30°C for 2 h in an orbital water bath shaker. The crude immobilized enzyme was washed after filtration to remove the unbound enzyme with glycine-NaOH buffer solution and subsequently dried through vacuum freeze-drying. In addition, the residual protein content in the enzyme solution was determined and used to estimate the fixation level of lipase in the enzyme immobilization. The fixation level (%) and the average protein concentration (mg/g) of the immobilized lipase were calculated according to a previously described method[23] to screen the optimal support.

$$Fixation\;level\;(\%)\;=\;\frac{P0\;-\;P1}{P0}\times \;100\%$$

$$Protein\;Content\;in\;the\;Resin\;(mg/g)\;=\;\frac{P0\;-\;P1}{S0\;+P0\;-\;P1}$$

Where P_0_ is the initial protein content (mg), P_1_ is the residual protein content after immobilization (mg) and S_0_ is the weight of immobilized resin (g). The optimal immobilization conditions were investigated after estimating the effects of the pH of the buffer solution (8.0, 8.5, 9.0, 9.5, 10.0, 10.5, 11.0 and 12.0), the initial protein concentration (10, 20, 30, 40, 50 and 60 mg/ml) and adsorbing time (0.5, 1.0, 1.5, 2.0, 2.5 and 3.0 h). Immobilization was performed in the same manner as used for the support screening process.

### Activity Assay for the Immobilized AOL

The synthetic activity was determined based on the acidolysis of palm stearin and OA. A total of 3.33 g of palm stearin and 9.17 g of OA (molar ratio 1:8) were added into a 100-ml round-bottomed flask and incubated at 65°C in a water bath shaker at 200 rpm. Subsequently, 1 g (8% of the total substrates weight) of the different types of tested lipases (immobilized AOL, RM IM, TL IM and AOL powder) was added to initiate the reaction, respectively, after the substrate mixture was homogenous. A total of 0.01 g of sample was regularly collected (0.25, 0.5, 0.75, 1.0, 1.5, 2.0 and 3.0 h) as the reaction proceeded and immediately dissolved into 1.0 ml of hexane for the product components analysis. In addition, before analyzing the composition of the FAs, the amount of free FAs in the reaction products was removed after neutralizing with excessive potassium hydroxide and rotary evaporation of the organic phase, according to the method described by Wei et al. (2015) [24].

To optimize OPO synthesis, the effects of the reaction conditions on enzymatic acidolysis and lipase specificity were investigated. The catalytic reactions were assessed using an immobilized lipase activity assay, examining the following reaction factors: reaction temperature (50, 55, 60, 65 and 70°C), molar ratio of substrates (1:2, 1:4, 1:6, 1:8 and 1:10), enzyme loads of immobilized lipase (4%, 6%, 8%, 10% and 12%) and the water content of immobilized lipase (3.5%, 15%, 30%, 40% and 50%). The lipase activity and specific activity were defined as the reaction rate divided by the quantity of immobilized enzyme and the quantity of protein, which were calculated using the following equations, in which 1 U of lipase activity was defined as 1 μmol C52 produced per minute at a certain temperature:

$$Lipase\;Activity\;(U/g)\;=\;\frac{M\Delta C52}{S1\;\times \;T}$$

$$Specific\;Activity\;(U/mg)\;=\;\frac{M\Delta C52}{P2\;\times \;T}$$

Where MΔC52 is the molar amount of C52 synthesized in the reaction (μmol); S_1_ is the weight of immobilized resin used (g); P_2_ is the protein content in the immobilized lipase (mg); and T is the reaction time for the synthesis of C52 (min).

### Analysis of acidolysis products through gas chromatography (GC)

C52, tripalmitin and diacylglycerols (DAGs) in the reaction products were detected through GC using an Agilent 7890A GC platform equipped with a flame ionization detector (FID) and a fused silica capillary column DB-1HT (15 m length × 0.25 mm internal diameter × 0.25 μm film thickness, Agilent, Santa Clara, USA). Nitrogen was used as the carrier gas at a flow rate of 20.0 ml/min. The column was initially maintained at 200°C, was gradually increased to 350°C at a constant rate of 8°C/min, and then was maintained at 370°C for 5 min. The detector temperatures were maintained at 370°C. Aliquots of 2 μl of the sample were injected in the split mode with a split ratio of 50:1. Standard calibration curves for C52 and tripalmitin were obtained after dissolving in hexane at gradient concentrations and analyzing through GC under the conditions described above. The standard curves were obtained from the relationship plot between the log peak area and log sample concentration.

### Regiospecific analysis of FAs in TAGs through GC

The FAs in all TAGs products were converted into the corresponding fatty acid methyl esters (FAMEs) using 2 mol/l KOH-methanol solution. The FAMEs analysis was performed according to the methods of Wei et al. (2015) with some modifications, using a 7890A GC platform (Agilent, Santa Clara, USA) equipped with a FID and a DB-23 column (30 m× 0.25 mm × 0.25 μm, Agilent, Santa Clara, USA). The column oven was initially maintained at 50°C for 2 min, gradually increased to 180°C at a rate of 10°C/min, and maintained it at 180°C for 5 min. The column was subsequently increased to 230°C at a constant rate of 5°C/min, and held at 230°C for 5 min. A 1-μl aliquot of sample was injected at a split ratio of 1:50. Supelco 37 Component FAME Mix (PA, USA) standard solution was used to identify chromatographic peaks and calculate the molecular weight correction factors of the individual FA peak areas.

The FA compositions at the sn-1,3 and sn-2 positions of TAGs products were analyzed to estimate the regiospecific distribution and the lipase regiospecificity. The regiospecific analysis of TAGs was performed according to Wei et al (2015). In general, 0.1 g of OPO was dissolved in 0.3 ml of hexane, and 2 ml of Tris-HCl buffer (1 M, pH 8.0), 0.2 ml of CaCl_2_ (220 g/l) and 0.5 ml of bile salts (1 mg/ml) were successively added while mixing. A total of 50 mg of PPL was introduced into the sample mixture, which was preheated in a water bath at 40°C with vigorous shaking, and incubated for 5 min. Subsequently, 1 ml of HCl (6 mol/l) and 2 ml of diethyl ether were added to terminate the reaction. The reaction mixture was divided into three layers after centrifugation, and the top layer with diethyl ether was separated through drying with anhydrous Na_2_SO_4_ and blowing with nitrogen.

The hydrolytic products were separated on silica gel GF 254 TLC plates, using n-hexane/diethyl ether/98% formic acid (70:30:1, v/v/v) as the developing solvent. The TLC plates were stained with 2, 7-dichlorofluorescein in ethanol (0.2%, w/v), and the sn-2 MAG band was carefully scraped from the plate and extracted using n-hexane. The FA composition of the sn-2 MAG was analyzed through GC using a previously described method. The FA contents at the sn-1,3 position were calculated after subtracting the corresponding FAs contents at the sn-2 position from the total concentration of the FAs in all TAGs. OA was introduced at the sn-2 and sn-1,3 positions through the acidolysis reaction were obtained and used to evaluate the sn-1,3 specificity of lipase (Ratio of sn-1,3 specificity, RS) using the following equation:

$${RS}_{sn-1,3\;specificity}\;=\;\frac{\Delta sn-1,3\;OA\%―\Delta sn-2\;OA\%\;}{\Delta sn-1,3\;OA\%+\Delta sn-2\;OA\%}$$

All tests and reactions in this study were performed in triplicate and the results were presented as the average.

## Results and Discussion

### Carrier Screening for AOL Immobilization

**Fig 1:**
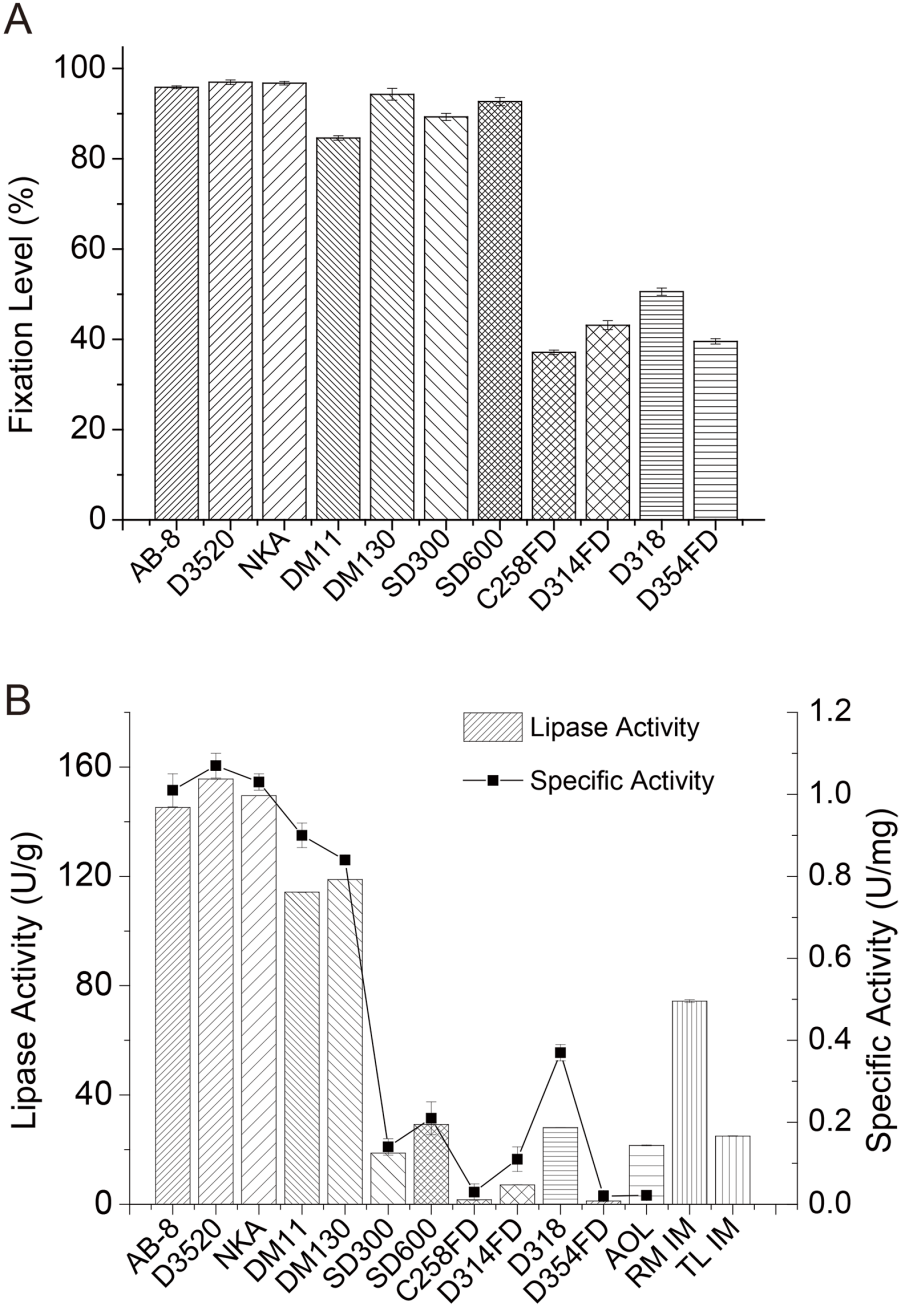
Carrier screening for immobilization of AOL: (a) Fixation level, (b) Lipase activity and specific activity of lipase in the acidolysis reaction for C52 synthesis from palm stearin and OA.

Lipase immobilization often contributes to the enhancement of the catalytic activity of this enzyme in nonaqueous system, and the effect of immobilization on lipase primarily depends on the method and the support material selected[16, 17]. The results of a previous report showed that the particles of 300 nm in diameter were suitable for immobilization[25]. Therefore, 7 PS-based non-polar and low polar resins and 4 ion-exchange resins of 300–1250 nm in diameter were screened for the immobilization of the sn-1,3-specific lipase AOL L03. Ethanol has been used to improve lipase immobilization on hydrophobic supports through decreasing the hydrophobicity of the inner surface of the pores and thereby increasing the access of lipase to these substances[17, 26]. All the hydrophobic supports displayed strong lipase adsorption, and the fixation levels were almost twice as high as that of the ion-exchange support (Fig 1A). D3520 had the highest fixation level (96.97%), and the DM11 was last among the hydrophobic supports (84.61%), with the smallest specific surface area (200–250 m^2^/g), and the fixation level of D318 (50.56%) was the highest among the ion-exchange supports. The immobilization yields are dependent on the structural and chemical characteristics of supports, and a large internal surface area is typically associated with high enzymatic activity.

The results of the acidolysis reaction catalyzed by the immobilized lipase or enzyme powder are shown in Fig 1B. AOL enzyme powder showed extremely low interesterification activity (21.6 U/g) and specific activity (0.02 U/mg), indicating that the powder could not be directly used in industry. Most immobilized lipases significantly improve the specific activity, among which the largest specific activity (D3520) showed a 49.54-fold increase, indicating that immobilization was particularly essential to the reaction and application of lipase in the nonaqueous system. Similar to the fixation level, both the activity and specific activity of lipases immobilized to hydrophobic supports showed better performances than those with ion-exchange supports. Among all the hydrophobic resins tested as lipase immobilizing carriers, AB-8, D3520 and NKA were the most effective, followed by DM130 and DM11, while SD300 and SD600 showed low efficiency lipase activity similar to the ion-exchange resins. Hydrophilic resin might compete and strip off the essential water from the lipase surface, resulting in a rigid and inflexible structure, thereby decreasing lipase activity. In addition, significant differences in the activity and specific activity among lipases immobilized with different resins contradicted the relatively similar results of the lipase fixation levels, indicating that lipase immobilization affected more than just the adsorption of a sufficient amount of the lipase in the particles[27, 28].

Although many studies have reported the successful application of ion-exchange supports in lipase immobilization through adsorption[19], hydrophobic supports are better candidates in most cases. The low specific activities of lipases in ion-exchange resins, partially reflected the fact that the enzymes are immobilized with the resins through a variety of chemical groups, ultimately disrupting contact between the substrate and the enzymes or even denaturing the enzymes through alterations in the protein conformation. In contrast, the improvement of the hydrophobic resins is likely due to several reasons. During immobilization, the lipase could be activated in the hydrophobic phase, and the open form could be stabilized through the hydrophobic interaction between the lipase surface around the active center and the matrices used for immobilization[29, 30]. Furthermore, the hydrophobic resins with local hydrophobic environments could increase the concentration of hydrophobic substrates, consequently increasing lipase activity[23]. However, lipases in SD300 and SD600, despite having high fixation levels, showed low catalytic activities in acidolysis reactions, suggesting that hydrophobic macroporous supports with a large internal surface area and a small pore diameter could adsorb large amounts of enzyme within the deep pore, where, unfortunately, the access of the substrate to the lipase is significantly restricted. These results illustrated that hydrophobic resins with a mean pore diameter between 8.0 and 22 nm were suitable to immobilize lipase for the catalytic synthesis of OPO-based structured lipids.

Because AOL immobilized in D3520, referred to as AOL D3520, showed the best efficiency in C52 synthesis, with 155 U/g acidolysis activity and 1.07 U/mg specific activity, even better than that of the commercial lipases RM IM (74.34 U/g) and TL IM (24.98 U/g) at the same particle weight, this enzyme was selected for use in further catalytic reactions.

### Optimization of AOL D3520 immobilization

#### Time course of AOL immobilization

**Fig 2:**
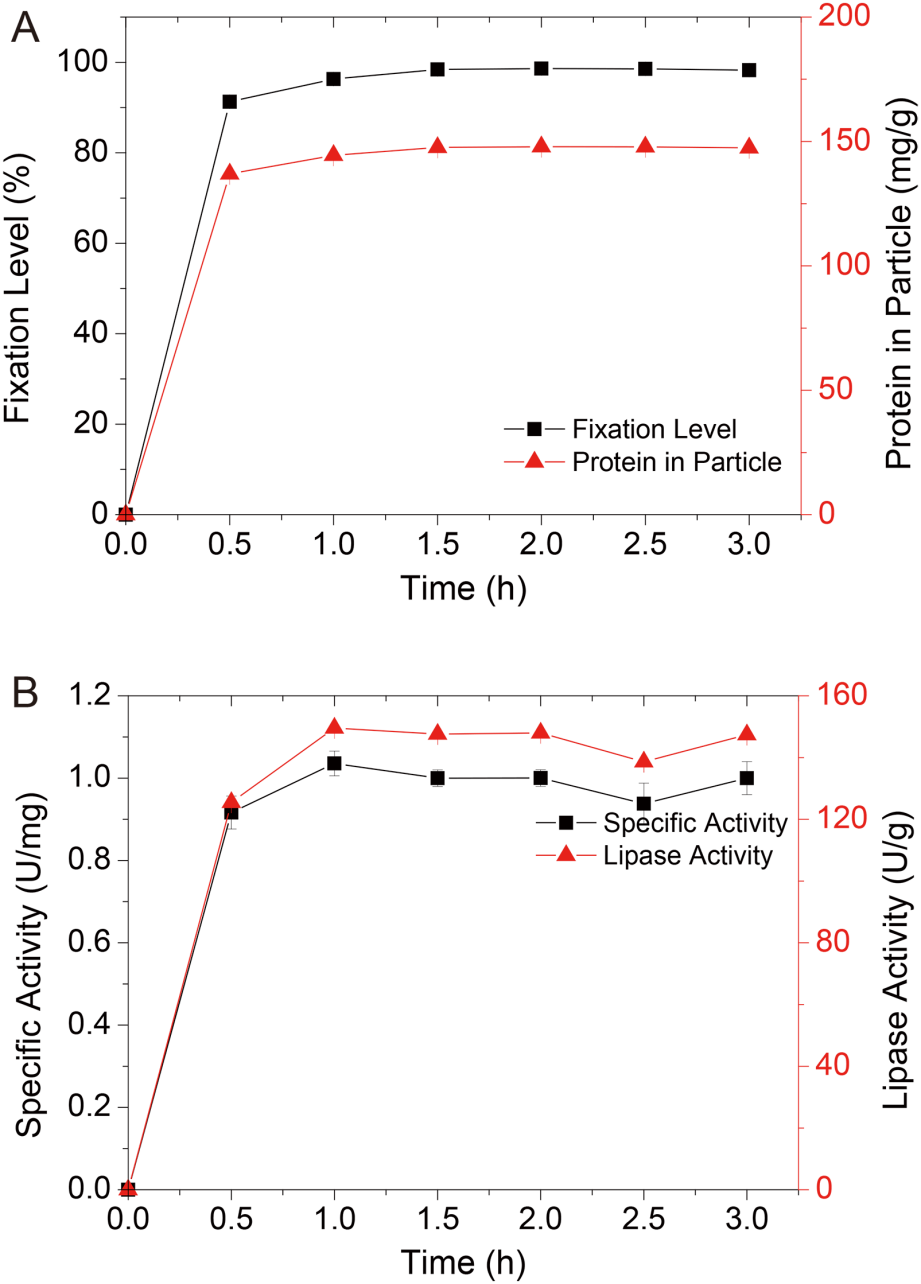
Effect of adsorption time on fixation level and protein amount in particle (a) and lipase activity and specific activity of AOL D3520 (b).

The interaction between the lipase and the carrier in lipase immobilization through adsorption is reversible[31], suggesting that the immobilized lipase was sensitive to variations in immobilization conditions. A time course for fixation and acidolysis during AOL D3520 immobilization was conducted. The results, shown in Fig 2A, demonstrated that the fixation level reached 91.29% in only 0.5 h and was slightly increased after 1.0 h. Adsorption at 1.5 h, with a fixation of 98.42%, lipase activity of 147.62 U/g and specific activity 1.00 U/mg, was considered as the time required for the completion of the adsorption of the enzyme into the support, demonstrating through the following plateau of fixation and catalytic efficiency. Although 1 h of adsorption showed slightly higher lipase activity and specific activity, this process was hypothesized as a combination transition state that might influence the stability of the catalyst. Consequently, 1.5 h was used as the immobilization time for further tests.

#### Effect of the protein concentration on AOL immobilization

**Fig 3:**
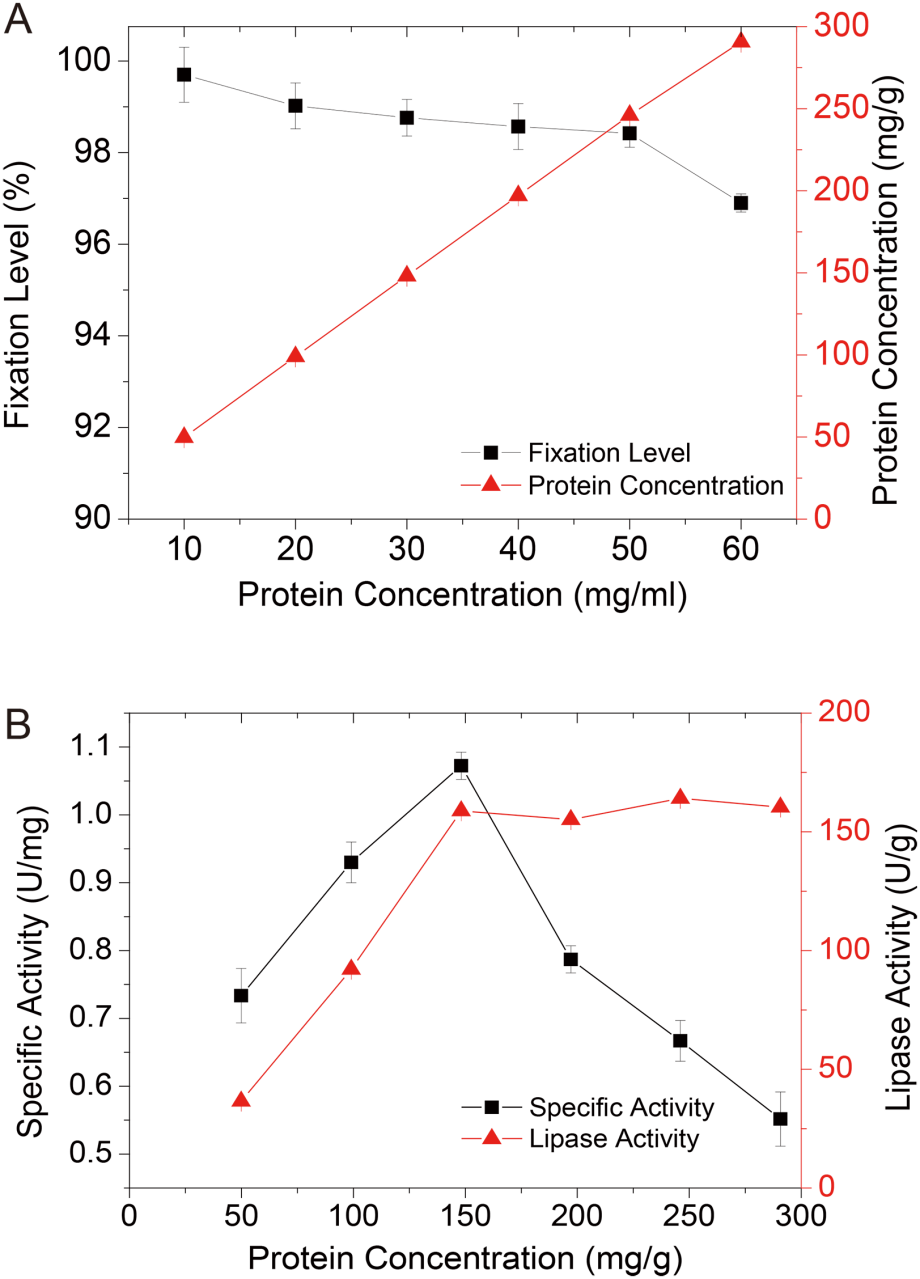
Effect of protein concentration on fixation level and protein amount in particle (a) and lipase activity and specific activity of AOL D3520 (b).

The initial protein concentration of the solution used for immobilization has been demonstrated as influencing both the fixation level and the lipase activity[23]. According to the results shown in Fig 3A, the protein amount adsorbed in the particles was tightly correlated with the original protein concentration of the solution between a scale of 10 to 60 mg/ml, corresponding to relatively high and constant fixation levels of lipase (96.9–99.7%), with only a slight decrease to 60 mg/ml of protein, suggesting that the D3520 resin showed increased adsorption for lipase immobilization. However, the result of lipase activity in the acidolysis reaction (Fig 3B) was different from the fixation level. As the lipase amount in the particles increased to approximately 150 mg/g, the lipase activity and specific activity reached 185.86 U/g and 1.07 U/mg, respectively, showing a strong upward tendency. Nevertheless, the increase in the amount of protein in the particles only slightly influences the lipase activity, resulting in a dramatic plunge of specific activity of the immobilized enzyme. The results further confirmed that the lipase amount was not the only factor that influenced the performance of the immobilized lipase.

In the present study, the specific activity was inhibited at both low and high protein concentrations in the particles. Previous studies have reported that, at a high concentration of lipase, the enzymes interface the active centers of two open forms and subsequently aggregate. Consequently, the lipase activity decreases because of the partial blockage of the active centers[27, 28]. In addition, the absorbance of the enzymes into the resins also accumulated in a multi-layer form[32], as there was no sufficient internal surface area for lipase binding. Moreover, the high protein amount might cause congestion in the internal pores of the resin, leading to the inaccessibility of the substrates to the lipases. In contrast, at a low protein amount, immobilized molecular lipase was unfolded, because of the interaction with the excess surface area of the matrix, and consequently partially inactivated[23]. To achieve more cost effective lipase immobilization, 148.14 mg/g of protein, corresponding to a 30 mg/ml initial protein concentration in the solution, was utilized for the following experiments.

#### Effect of the buffer pH on AOL immobilization

**Fig 4:**
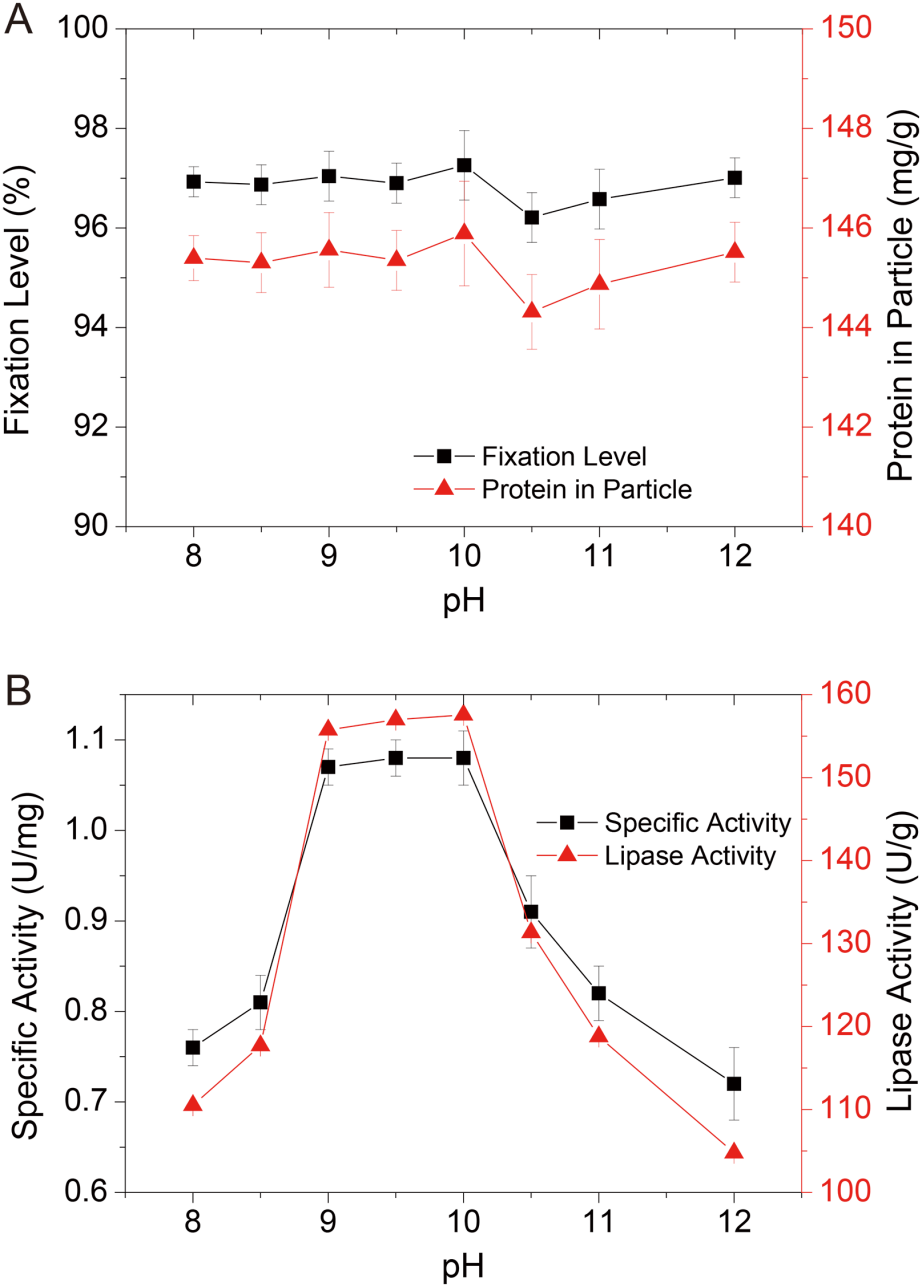
Effect of pH on fixation level and protein amount in particle (a) and lipase activity and specific activity of AOL D3520 (b).

The pH was also one of the most important factors in enzyme immobilization, as this parameter influences not only the combination between the enzymes and matrices but also the molecular conformation and structure of the lipase in the final catalyst. The lipase fixation level fluctuated on a smaller than 1% scale as the pH varied between 8 to 12, and peaked to 97.26% at pH 10 (Fig 4A). The limited effect indicated that the carrier amount was sufficient to exclude the influence of solution pH on adsorption of lipase. Based on little variation in the adsorption of the protein amount in the particles with increasing pH, the immobilized lipase activity and specific activity varied in catalyzing the acidolysis reaction. When the pH of the lipase immobilization solution was between 9 and 10, optimal catalytic activity was observed, showing an apparent decline in activity when the pH exceeded this range. The optimum pH range of AOL for the acidolysis reaction only slightly shifted compared with that of the lipase from *A*. *oryzae* for the hydrolytic reaction as previously reported [33], displaying optimal activity at a pH between 8 to 9, and high levels of activity between pH 7 and 10. These results confirmed that the molecular conformation of lipase under immobilization conditions could be reserved in the particles in the organic environment[17, 34], referred to as “molecular memory”[35], suggesting that enzyme immobilization optimization was one of the efficient measures for improving the properties of lipase as the industrial catalyst.

### Factors influencing the sn-1,3 specificity of the immobilized lipase

**Fig 5:**
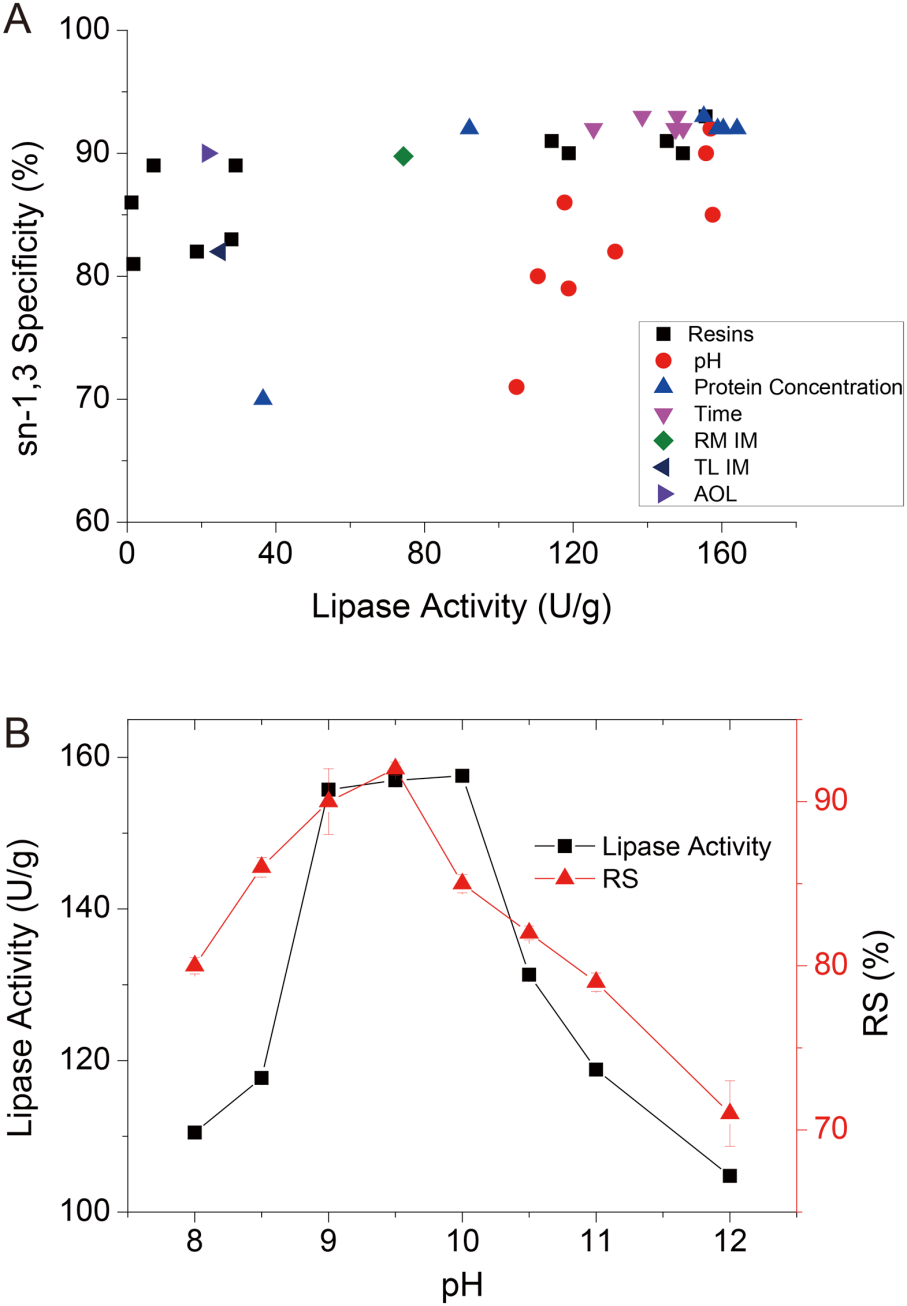
Relationship analysis between lipase activity and sn-1,3 specificity: (a) relationships between the lipase activities of different immobilized lipases and their sn-1,3 specificities; (b) influence of pH on the lipase activity and sn-1,3 specificities.

The lipid content of structured OPO, enzymatically converted from the acidolysis reaction between palm stearin and OA, was mainly determined based on the C52 content and sn-1,3 specificity of the lipase. The sn-1,3 specificity of the lipase was estimated according to the relationship between the OA introduced at sn-1,3 and sn-2 positions through the acidolysis reaction previously described herein. In the present study, the lipase catalysts from different immobilization resins, and those with different times, protein concentrations and solution pH values were investigated for sn-1,3 specificity. The lipase activity displayed in the reaction was plotted on the x-axis, and the sn-1,3 specificity of the lipase was plotted on the y-axis (Fig 5A). The immobilized lipases and AOL powders showed a high level of sn-1,3 specificity in the acidolysis reaction, with values higher than 80%. In addition, lipase sn-1,3 specificity was highly positively correlated with the enzymatic activity, with a Spearman’s rho of 0.74 (P < 3.34 × 10^−5^) and Pearson’s linear correlation of 0.81 (P < 1.71 × 10^−6^), determined after the exclusion of some deviated data for the pH and protein concentration. This result could partially reflect the internal acyl migration reactions, which played a major role in the quality deterioration of structure lipid production[36]. As the lipase displayed higher activity in the reaction, the migration of the OA content from the sn-1,3 position to the sn-2 position would comprise less in proportion to all the introduced OA at sn-2 position, and the effect of acyl migration was diluted, resulting in an increased RS value or sn-1,3 specificity of lipase.

After calibration with the relationship, these immobilized lipases showed sn-1,3 specificity analogous with that of RM IM and TL IM, immobilized forms of the typical sn-1,3-specific lipases RM and TL frequently used in the enzymatic synthesis of structured lipids[8]. Although previous studies have suggested that the immobilization of lipases into hydrophobic supports could potentially change lipase specificity towards hydrophobic substrates[28, 37], the results of the present study showed that variations in the sn-1,3 specificity of the immobilized lipase for hydrophobic resins primarily reflect differences in the acyl migration resulting from different lipase activities. Similar results were observed for lipase catalysis at different times, indicating that the sn-1,3 specificity of lipase was not affected by the time course.

However, a low concentration (10 mg/ml) of immobilized lipase showed abnormally low sn-1,3 specificity, potentially reflecting the strong interaction between the hydrophobic support and the protein, thereby decreasing the catalytic activity and specificity activity. The pH value of the immobilization buffer used in the present study was the most striking factor that changed the sn-1,3 specificity of the AOL D3520. Fig 5B showed that the curve of lipase activity in response to pH was not consistent with that of the sn-1,3 specificity of lipase. Under alkaline conditions, the regiospecificity of lipase increased as the pH increased to 9.5 and decreased with increasing pH. This result indicated that some partial protein configuration was slightly changed, particularly in the alkaline amino acid residues of the protein, as their charging states were potentially more influenced by pH variations in the alkaline range. This result suggests that the substrate pocket for the sn-2 chain is more hydrophilic compared with that for the sn-1,3 chain[38, 39], implying that pH variation might change the hydrophilic amino acids associated with the pocket for the sn-2 chain. In addition, a previous study reported that the catalytic His residue plays a role in determining the stereospecificity of *Pseudomonas cepacia* lipase[40]. An additional report showed that stereopreference can be estimated after determining the width and shape between the side chains of the His gap residues, showing a hydrophobic dent hosting the sn-2 chain of TAGs or analogs[41]. For these studies, we inferred that the effect of pH on lipase regiospecificity, tightly related to stereospecificity, primarily reflected the charge state variation of alkaline amino acid residues associated with the substrate pocket for the sn-2 chain, particularly the catalytic His residue of lipase.

### Effect of temperature on C52 synthesis

In addition to the high sn-1,3 specificity of the biocatalyst AOL D3520 immobilized under optimal conditions, the synthesized amount of OPO in a stable system was determined based on the C52 yield. DAG production was detected and used as an indicator of the side reaction. A high reaction temperature was required to dissolve and homogeneously mix the substrates OA and palm stearin, with a 58°C melting point. Furthermore, the higher temperatures would increase the mass transfer and catalytic efficiency of the biocatalyst. In contrast, higher temperatures might increase the energy consumption and side effects, such as fat oxidation, and be detrimental for the operational stability of the reaction system with a heat-sensitive lipase. Higher reaction temperatures also enhanced the internal migration of acyl groups in the structured TAG and thus reduced the expected OPO yield. Previous studies have demonstrated that the solvent system for OPO synthesis could decrease the temperature of the acidolysis reaction and obtain a comparable product yield[24], however, with adverse consequences on production separation and the economic and environmental burden.

**Fig 6:**
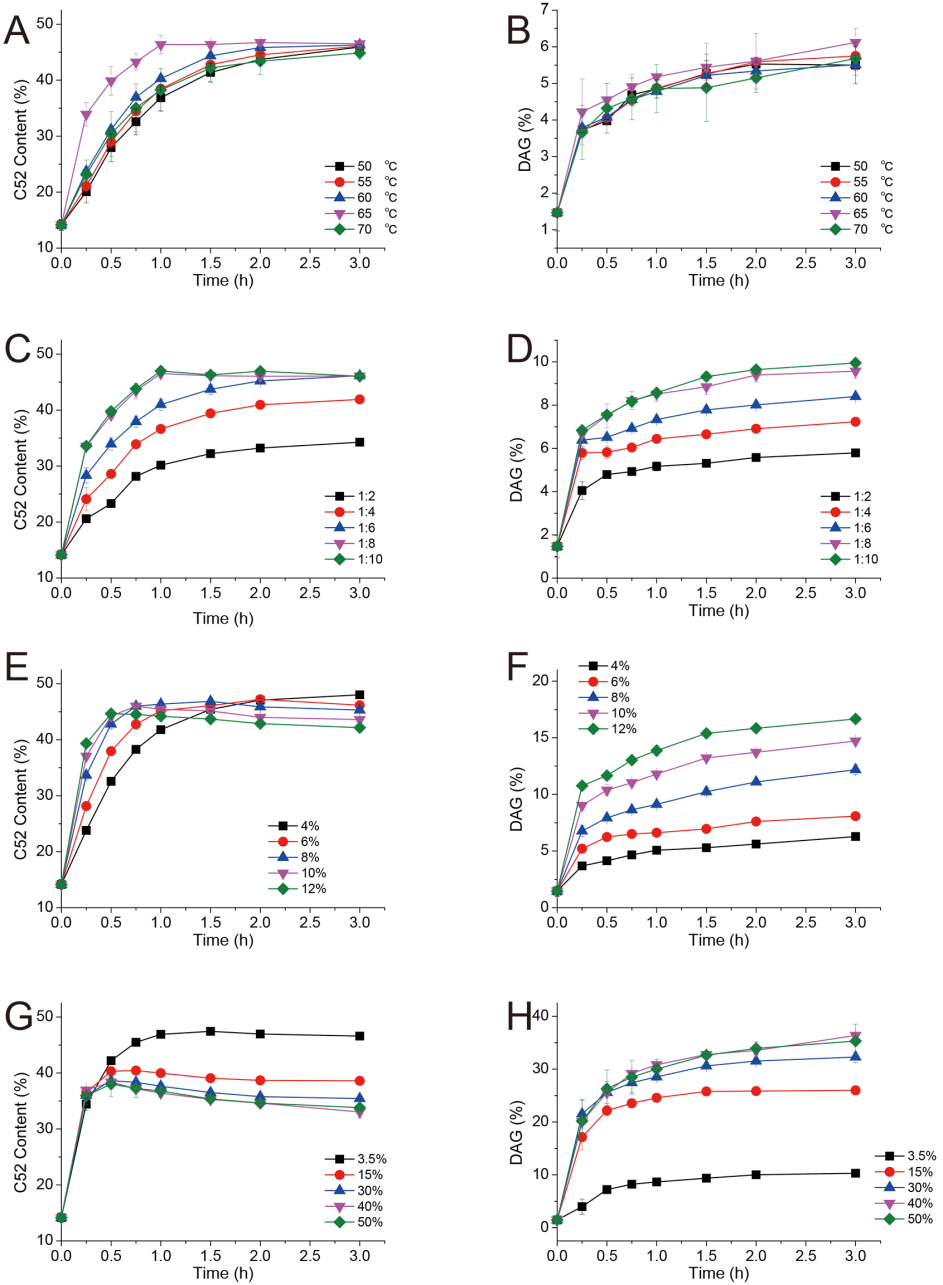
Effect of the reaction conditions on the synthesis of C52 and DAG: the effect of the reaction temperature, molar ratio, enzyme loading and water content on the synthesis of C52 (a, c, e, g) and DAG (b, d, f, h).

The effect of temperature on C52 synthesis in the solvent-free system in time was determined, and the results are shown in Fig 6A. The C52 content curves obtained at different temperatures were initiated with upward slopes, successively plateauing one after the other. All C52 yields after 3 h were close (approximately 46%), indicating that the differences in the lipase activity could be offset after extending the time, and the final yields were primarily determined based on the composition and purity of the original lipid material when time was sufficiently long. The initial lipase activity and C52 production before the peak point increased with the increasing reaction temperature until 65°C, and rapidly decreased at 70°C, suggesting that a temperature of 65°C was optimum for efficient OPO synthesis. Surprisingly, the DAG production at the different temperatures was analogous (shown in Fig 6B), and the DAG amount was 5.5–6.0% after 3 hours, implying that DAG synthesis was slightly influenced by the reaction temperature.

### Effect of the molar ratio of the substrates on C52 synthesis

It was ideal to use twice the amount of OA and tripalmitin in molar quantity to synthesize OPO according to the reaction formula. However, the acidolysis reaction was reversible and regulated through the feedback of the products. In addition, the complex substrate materials and the incomplete regiospecificity of lipase introduced a series of side reactions in the system, resulting in the infeasibility of the complete conversion for all substrates. The optimal molar ratio of the substrates for the highest C52 yield was a typical compromised strategy, identified through the effect test of different molar ratios (shown in Fig 6C). In response to the increased molar ratio of OA and palm stearin from 2:1 to 6:1, the amount of converted C52 peaked (46.14%) with a decreasing slope, and remained at a maximum level with increasing molar ratio to 10:1. Nevertheless, C52 synthesis peaked in a shorter time at a higher molar ratio (8:1 and 10:1). The curves of C52 yield at the molar ratios of 8:1 and 10:1 were nearly identical, indicating that increasing the amount of OA would not necessarily improve the C52 yield. In addition, increasing the molar ratio would persistently increase the DAG production to 9.95% (Fig 6D), with a decreasing acceleration, reflecting the introduction of water from the increased OA (0.3% water content). Thus, improve catalytic and economic efficiency, an optimum molar ratio of 8:1 between OA and palm stearin is required.

### Effect of catalyst loading on C52 synthesis

A large quantity of catalyst load could benefit the structured lipid synthesis by shortening the reaction time and weakening the effect of acyl migration. The increased enzyme loading in the present study increased the initial rate of C52 synthesis (shown in Fig 6E). As enzyme loading increased, the amount of C52 decreased in a shorter time, with a lower final yield after 3 h. An enzyme load of 8% (w/w) generated the largest amount of C52 (46.87%) after 1.5 h, and showed preferable yield and catalytic efficiency.

Correspondingly, the amount of DAG increased with increased enzyme loading (shown in Fig 6F). This result might reflect the high water content in the immobilized lipase (3.5%), resulting in the hydrolysis of TAGs to the corresponding DAGs. Therefore, it was necessary to dry the prepared catalyst to examine the lowest essential water, as the acidolysis reaction was apparently sensitive to the water content. An 8% (w/w) enzyme load showed a better comprehensive performance; therefore, this amount was selected for the final reaction system.

### Effect of water content on C52 synthesis and the side reaction

Most enzymes become more stable in organic environments, but a low water content on the lipase surface is essential for maintaining the necessary flexibility of protein structure and catalyzing the acyl transfer reactions[35, 42, 43]. In addition, a high water content in the synthetic reaction of TAG will definitely result in hydrolytic side reactions; thus, the water content of the catalytic environment markedly influences the lipase activity and product composition[44]. The prepared immobilized lipase adsorbed 3.5% water and showed high DAG production in the reaction; therefore, we further determined the influence of the high water content on the acidolysis reaction. The results, shown in Fig 6G and 6H, were consistent with the effect of the increasing water content from the loading of the immobilized lipase. The peak yield of C52 decreased with increasing water contents to 40%, and the DAG production correspondingly increased. Surprisingly, the 40% and 50% water content in the acidolysis system showed similar results in the C52 and DAG amount as time elapsed, implying a kinetic equilibrium between transesterification, esterification and hydrolysis at these levels of water content. Because the activity gap between transesterification and esterification has been previously reported for other lipases[45–47], we hypothesized that this effect might explain the offset of increased esterification activity, compared with the relatively weak transesterification activity, to the increasing hydrolytic reaction catalyzed through the AOL D3520 catalyst involving increasing water as the substrate.

Overall, the optimal OPO synthetic conditions (temperature, 65°C; substrate molar ratio, 1:8; enzyme load, 8% (w/w); water content, 3.5%; reaction time, 1 h) generated 45.65% C52, with a tripalmitin content of 2.75% in the system and a sn-2 PA proportion of 55.08%. This result was adequate for industrial manufacture and comparable with previous results[6, 18, 48].

## Conclusion

In summary, the sn-1,3-specific lipase AOL was highly and efficiently immobilized with the PS-based hydrophobic resin D3520, showing a significant 49.54-fold increase in specific lipase activity compared with the AOL powder. The optimal immobilization conditions were investigated, including the time course, initial protein concentration and solution pH. The sn-1,3 specificities of lipases under different immobilization conditions were evaluated and demonstrated as positively correlated with the lipase activity, potentially reflecting acyl migration, and the pH of the immobilization solution was shown to influence lipase regiospecificity and synthetic activity.

The prepared immobilized AOL D3520, as a biocatalyst, was used for the enzymatic synthesis of structured lipid OPO through acidolysis between palm stearin and OA. The optimal conditions for the synthesis of structured lipid OPO were investigated, and the following condition were observed: 65°C temperature; 1:8 substrate molar ratio between palm stearin and OA; 8% (w/w) enzyme load; 3.5% water content of the immobilized lipase; and 1.5 h reaction time. Under these conditions, highly efficient C52 production (45.65%), with a tripalmitin content of 2.75% and a sn-2 PA proportion of 55.08%, was achieved.
